# The Fourth Annual Report of the State Board of Health of Illinois

**Published:** 1883-02

**Authors:** 


### THE FOURTH ANNUAL REPORT OF THE STATE BOARD OF HEALTH OF ILLINOIS.

This report reveals the fact that the State of Illinois is favored with the most useful and effective State sanitary organization, so far as our knowledge goes, in America. This Board is not only charged with the duty of protecting the sanitary interests of the State, but it also has the power to regulate the practice of medicine within the commonwealth, by revising all medical diplomas and issuing licenses to such persons only who, in the judgment of the Board, are qualified to practice medicine, and by revoking any license when it appears that the holder is guilty of unprofessional or dishonorable conduct.

The statutes are ample; the organization is admirable; and the execution of the law seems to be perfect.

A large measure of the success of this Board is due to the untiring zeal, the unflinching fidelity and the undoubted ability of its executive officer, the energetic Secretary. Here the man and the occasion have met; and in this volume we find recorded the legitimate fruits of the union.

In view of the importance of this whole subject, both to the medical profession and to the general public, we shall, in our next issue, give an extensive review of this question as expounded and illustrated in the report and in the workings of the Illinois Board.

This example is worthy of imitation in other States.

